# Medial collateral ligament reconstruction using bone-patellar tendon-bone allograft for chronic medial knee instability combined with multi-ligament injuries: a new technique

**DOI:** 10.1186/s13018-016-0416-8

**Published:** 2016-07-22

**Authors:** Xiaozuo Zheng, Tong Li, Juan Wang, Jiangtao Dong, Shijun Gao

**Affiliations:** Department of Orthopedics, Third Hospital of Hebei Medical University, 139 Ziqiang Road, Shijiazhuang, 050051 Hebei People’s Republic of China; Orthopaedic Biomechanics Laboratory of Hebei Province, 139 Ziqiang Road, Shijiazhuang, 050051 Hebei People’s Republic of China

**Keywords:** Knee, Medial collateral ligament, Reconstruction, Technique

## Abstract

**Background:**

The medial collateral ligament (MCL) is the main static stabilizer of the medial knee. The surgical treatment was recommended in cases with serious medial collateral ligament insufficiency combined with multi-ligament injuries and chronic symptomatic medial instability. Several surgical techniques have been described for the MCL reconstruction, while potential problems including donor site morbidity, complicated procedure, and high risk of femoral tunnel collision were reported. In order to minimize such potential limitations, we describe a new medial reconstruction technique for MCL injury using bone-patellar tendon-bone (BPTB) allograft.

**Methods:**

A longitudinal incision at the medial knee was made. The centers of femoral and tibial attachments were gained through repeated isometricity test. Then, the bone grooves were made around the femoral and tibial centers. The appropriate BPTB allograft was selected, and both ends were trimmed. The prepared bone blocks were embedded into the grooves and fixed with cancellous screws. The programmed rehabilitation exercises were performed after the operation.

**Results:**

A strong graft and bone-to-bone healing on both femoral and tibial attachment sites were obtained, and femoral tunnel collision during multi-ligament reconstruction was avoided. Satisfactory valgus and rotatory stability were gained.

**Conclusions:**

This novel MCL reconstruction technique using BPTB allograft can be safely performed, and the clinical outcome was favorable with satisfactory valgus and rotatory stability. More cases and additional follow-up results are needed to verify the overall effect of this technique.

## Background

The medial collateral ligament (MCL) is the main static stabilizing structure against valgus and rotation of the knee. It is a broad, flat, and long ligamentous tissue, which originates around the medial femoral condyle and inserts on the proximal medial tibia. Most MCL injuries could be treated nonoperatively with good clinical outcomes because of the strong healing capacity [1, 2]. However, surgical treatment was recommended in cases with serious MCL insufficiency combined with multi-ligament injuries and chronic symptomatic medial instability [3–5]. In chronic cases with serious medial knee instability, there is little chance that the MCL will heal well and restore valgus stability [6]. Therefore, surgical treatment would be necessary.

Several surgical techniques have been described for the MCL reconstruction, such as direct repair [7, 8], proximal advancement of MCL [9], isolated MCL reconstruction [10, 11], and anatomical MCL reconstruction using a double-bundle technique [4, 12]. However, potential problems with these surgical techniques were reported. Yoshiya et al. argued that the autogenous semitendinosus and gracilis tendons graft harvest in the MCL reconstructive procedure may result in a loss of function of the pes anserinus tendons which serve as secondary medial stabilizers [10]. Dong et al. considered that augmented repair was not a good choice for the subacute MCL injury, and simple repair may not be as reliable as surgical reconstruction [8]. Moreover, in cases of simultaneous posterior cruciate ligament (PCL)-MCL reconstruction, two tunnels are needed to be created in the same condyle and close to each other. In this circumstance, high risk of tunnel collision was found during the surgical procedure [13].

In order to minimize such potential limitations, we describe a simple reconstruction procedure of MCL for chronic medial knee instability using bone-patellar tendon-bone allograft. The rationale of this technique is to obtain a strong graft and bone-to-bone healing on both femoral and tibial attachment sites and to avoid femoral tunnel collision during multi-ligament reconstruction.

## Surgical procedure

A 50-year-old man suffered a traumatic accident and was diagnosed with anterior cruciate ligament (ACL), PCL, and grade III MCL injuries of the right knee. The patient underwent simultaneous ACL and PCL reconstruction, combined with MCL anatomic repair at the time of surgery as acute injuries. While residual medial laxity was still obvious 3 months after direct repair and we believed that a reconstructive procedure was needed, with the patient under general or epidural anesthesia, the physical examination was performed to confirm MCL laxity that required reconstruction. A routine diagnostic arthroscopic evaluation was carried out and intra-articular damages were treated. As expected, continuity was seen on any of the reconstructed grafts, and subjective tension of either graft with a probe was marked taut.

**Fig. 1:**
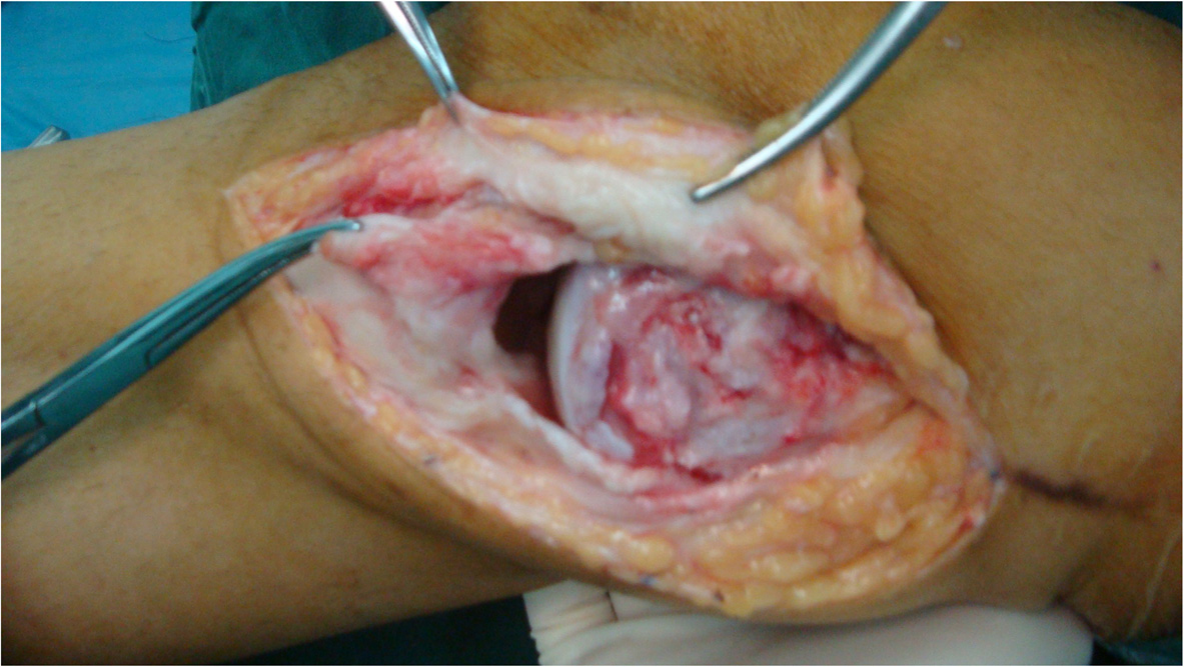
A longitudinal incision was made, and the severity of MCL damage was evaluated

After the arthroscopic procedure, the following reconstruction steps were carried out. A longitudinal incision was made from 1 cm above the medial femoral epicondyle to the insertion of the pes anserinus. (In this case, the incision previously made for repairing MCL was used). The medial structures of the knee were assessed under direct visualization. The medial femoral epicondyle and pes anserinus were identified as the landmarks of the femoral and tibial attachments of superficial MCL. The repair began from the deepest structures to the most superficial structures (Fig. 1). Interrupted absorbable suture method was used for repairing, if needed.

**Fig. 2:**
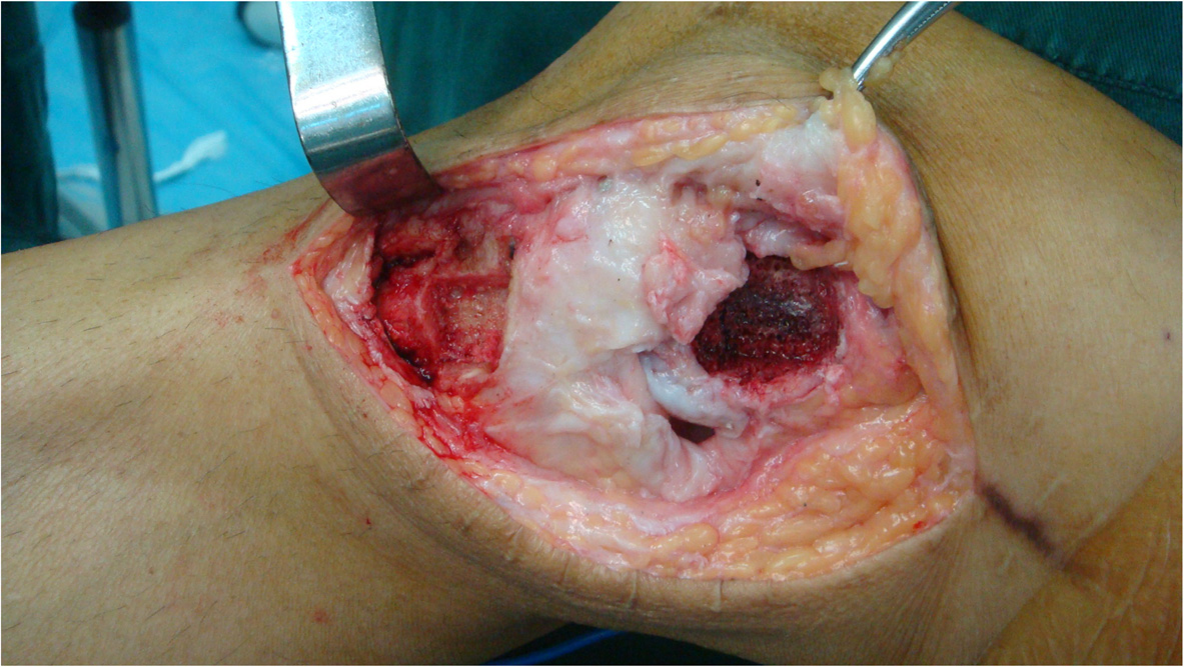
The bone grooves were made around the centers of the femoral and tibial attachments after satisfactory isometricity was gained through repeated modification

A 2-mm guide pin was drilled at the center of the femoral attachment of the MCL, which was located 3–5 mm proximal and posterior to the medial epicondyle. The estimated tibial insertion was located about 4.5 cm below the tibial plateau. An isometric test was performed through a 0–90°knee motion. The femoral and tibial insertion points were modified until satisfactory isometricity; a less than 2-mm length change during knee movement was gained. Once the isometric points were determined, the bone grooves were made around the centers of the femoral and tibial attachments. The femoral portion was about 10 mm in length, 10 mm in width, and 3 mm in depth. The tibial potion was about 20 mm in length, 10 mm in width, and 3 mm in depth (Fig. 2).

**Fig. 3:**
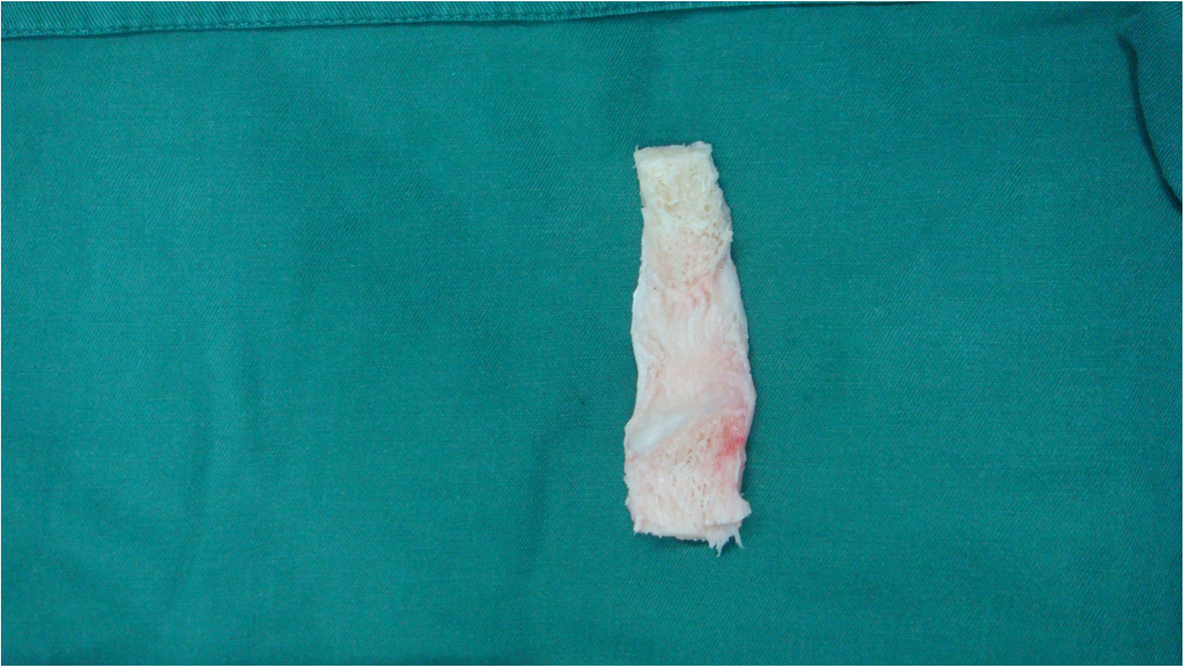
Bone-patellar tendon-bone (BPTB) allograft was selected, and both ends were trimmed to match the bone grooves

**Fig. 4:**
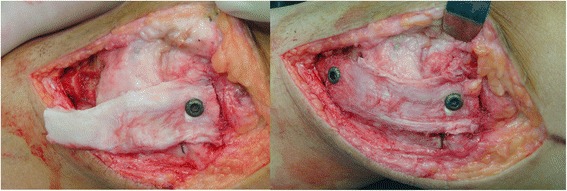
The femoral and tibial bone blocks were embedded into the grooves and fixed with cancellous screws

The length between the femoral and tibial grooves was measured with the knee placed at 30°of flexion with varus stress and neutral rotation. The appropriate bone-patellar tendon-bone (BPTB) allograft was selected according to the length of measurement. Both ends were trimmed into rectangular shapes to match the bone grooves (Fig. 3). Then, the femoral and tibial bone blocks were embedded into the grooves and fixed with cancellous screws, with the knee at 30° of flexion with varus stress and neutral rotation (Fig. 4). If possible, the torn posterior oblique ligament (POL) was identified and repaired with interrupted absorbable sutures. The anterior aspect of it was sutured to the posterior region of the reconstructed MCL. After all procedures were completed, a gentle valgus test was performed to ensure that the stability of the MCL reconstruction was adequate.

Postoperatively, a long hinged brace locked in extension was used for 2 weeks. The patient was encouraged to do isotonic exercise for quadriceps and hamstrings 48 h after the surgery. For isolated MCL reconstruction, knee motion of 30°–90°in the brace was allowed from four to six postoperative weeks. A goal of 120° flexion was expected at 8 weeks postoperatively. Partial and full weight-bearing exercises were allowed 6 to 12 weeks after surgery, and the brace should be weaned off gradually. Free activity was allowed after 3 months, and return to sports was not permitted until 6 months postoperatively. For combined MCL-ACL reconstruction, a similar rehabilitation plan was applied, but return to sports should be delayed to at least 9 months after the operation. For combined MCL-PCL reconstruction, more restrictive rehabilitation plan was applied. The brace wearing time should last for at least 3 months. Partial weight-bearing exercise should be delayed to 8 weeks postoperatively, and the brace could not be weaned off until 12 weeks postoperatively. Return to sports was not allowed until 12 months after the surgery.

## Discussion

Most of the grafts used for MCL reconstruction are tendon-like tissue, and the femoral and tibial attachments are point-like which were fixed with interference screws. However, the anatomical studies have demonstrated that the superficial MCL is a broad, flat, and long ligamentous tissue and almost composed of parallel fibers [14, 15]. The femoral and tibial attachments were broad-based, with areas 79.7 and 348.6 mm^2^, respectively. The width of the superficial MCL was about 10 to 17 mm from the proximal and distal ends to the middle part [15]. We do not think the tendon-like graft could provide sufficient strength compared with uninjured MCL in the early period of ligament reconstruction, although biomechanical studies are needed to prove our hypothesis. In our technique, BPTB was used as graft for reconstruction of MCL. The first advantage was that the BPTB graft could reproduce the shape of superficial MCL more anatomically because it was also a broad, flat, and long ligamentous tissue with two large insertion ends. More importantly, the graft offers sufficient strength for restoring knee kinematics and stability compared with single and thin tendon graft. Secondly, the BPTB graft provided two cancellous bone ends and that allows bone-bone healing at both femoral and tibial attachments, which are more reliable than tendon-bone healing.

Simultaneous MCL-ACL and MCL-PCL injuries are very common. During these multi-ligament reconstruction procedures, two or more femoral tunnels were required for graft placement. In cases of simultaneous MCL-ACL reconstruction, no femoral tunnel collision would happen because of different condyle tunnel placement. While, in cases of simultaneous MCL-PCL reconstruction, high risk of femoral tunnel collision was found because two tunnels were created in the same condyle and close to each other [13], in our surgical procedure, no femoral tunnel was needed for MCL reconstruction procedure. Only a 3-mm-depth bone groove was created under direct vision, and the graft placement was relatively simple. A guide pin was firstly initiated under fluoroscopy to avoid screw-PCL tunnel collision and that allows secure fixation on the femoral end. By taking this technique, we could simultaneously reconstruct MCL and PCL safely and effectively.

Several double bundle techniques have been described to restore medial knee stability. Many authors suggested superficial MCL reconstruction combined with POL reconstruction for chronic medial laxity. The POL is a condensation of the posteromedial joint capsule located posterior to the superficial MCL [16] and serves as a primary restraint to the rotation and a secondary restraint to valgus translation [4]. While whether concomitant superficial MCL-POL reconstruction could better improve both valgus and rotation stability compared with isolated MCL reconstruction is still debated [17] and the indication for reconstruction of the POL in the treatment of medial knee injuries requires further research, previous study argued that the superficial MCL also played an important role in maintaining rotational stability [8]. It could effectively restore favorable kinematics and stability in the superficial MCL and POL deficient knee by using a broad graft [6, 17]. From a mechanical view, a quadrangular structure could provide rotatory stability to some extent. We do think this broad quadrangular BPTB graft used in our technique could provide enough tissue and sufficient strength for restoring both valgus and rotational stability. Further biomechanical test and case-series studies are required to evaluate the outcome of this technique.

For medial knee injuries, controversies still exist over that how to choose the best treatment reasonably. It was generally accepted that isolated low-grade, partial medial collateral ligament injuries (grade I and II) could be treated nonoperatively [18]. While the surgery was recommended in cases of complete medial knee injuries (grade III) or with concomitant cruciate ligament injuries [18], some surgeons have recommended MCL reconstruction rather than surgical repair for chronic phase because poor-quality scarring and incomplete healing of the MCL are very difficult to identify and repair of those structures could hardly yield satisfactory results [7, 8, 19]. In addition, in cases of complete medial knee injuries combined with cruciate ligament injuries, MCL reconstruction was more reliable than simple repair to restore knee stability [8]. Thus, this MCL reconstruction technique was recommended in the following situations: (1) chronic grade III MCL injury with symptoms of medial instability, (2) acute medial knee injury combined with cruciate ligament injury that need simultaneous reconstruction, and (3) failure of MCL repair or reconstruction that need revision.

Certainly, there are some limitations in our MCL reconstruction technique. Firstly, this new technique may not reconstruct the superficial MCL anatomically because of the attachment sites of the reconstructed MCL depending on the length of BPTB allograft. While the isometric point should be gained through repeated modifications, the medial stability could be restored satisfyingly in knee flexion and extension positions. Secondly, two metallic screws were used to fix the graft. Friction at the skin-screw interface may lead to pain and tenderness in the medial part of the knee. Thus, an additional operation to remove the internal fixation was recommended when the bone healing was achieved. In addition, potential weakness in the present study was that the number of patients was limited, short-term follow-up period, and we also did not evaluate postoperative performance of the patients.

## Conclusions

This novel MCL reconstruction technique using a broad and strong BPTB allograft provides bone-to-bone healing on both the femoral and tibial attachments. More importantly, it could provide satisfactory valgus and rotatory stability and avoid femoral tunnel collision during multi-ligament reconstruction. More cases and additional follow-up results are needed to verify the overall effect of this technique.

## Abbreviations

ACL, anterior cruciate ligament; BPTB, bone-patellar tendon-bone; MCL, medial collateral ligament; PCL, posterior cruciate ligament; POL, posterior oblique ligament
